# The impact of regulatory T cells on the graft-versus-leukemia effect

**DOI:** 10.3389/fimmu.2024.1339318

**Published:** 2024-04-22

**Authors:** Carolina P. Pacini, Maria V. D. Soares, João F. Lacerda

**Affiliations:** ^1^ Instituto de Medicina Molecular João Lobo Antunes, Faculdade de Medicina, Universidade de Lisboa, Lisbon, Portugal; ^2^ Serviço de Hematologia e Transplantação de Medula, Hospital de Santa Maria, ULS Santa Maria, Lisbon, Portugal

**Keywords:** graft-versus-leukemia, regulatory T cell, immunomodulation, GvHD, cell therapy, hematopoietic stem cell transplantation

## Abstract

Allogeneic Hematopoietic Stem Cell Transplantation (allo-HSCT) is the only curative therapy for many hematologic malignancies, whereby the Graft-versus-Leukemia (GVL) effect plays a pivotal role in controlling relapse. However, the success of GVL is hindered by Graft-versus-Host Disease (GVHD), where donor T cells attack healthy tissues in the recipient. The ability of natural regulatory T cells (Treg) to suppress immune responses has been exploited as a therapeutical option against GVHD. Still, it is crucial to evaluate if the ability of Treg to suppress GVHD does not compromise the benefits of GVL. Initial studies in animal models suggest that Treg can attenuate GVHD while preserving GVL, but results vary according to tumor type. Human trials using Treg as GVHD prophylaxis or treatment show promising results, emphasizing the importance of infusion timing and Treg/Tcon ratios. In this review, we discuss strategies that can be used aiming to enhance GVL post-Treg infusion and the proposed mechanisms for the maintenance of the GVL effect upon the adoptive Treg transfer. In order to optimize the therapeutic outcomes of Treg administration in allo-HSCT, future efforts should focus on refining Treg sources for infusion and evaluating their specificity for antigens mediating GVHD while preserving GVL responses.

## Introduction

Graft-versus-Leukemia (GVL) is a crucial aspect of the success of Allogeneic Hematopoietic Stem Cell Transplantation (allo-HSCT) in the treatment of hematologic malignancies. This effect relies on the ability of donor T cells to recognize and eliminate tumor cells in the recipient’s body. However, this potent immune response can also lead to Graft-versus-Host Disease (GVHD), where donor-derived T cell clones react to antigens within healthy tissues of the recipient. The fine balance between GVHD and GVL responses is key for long-term leukemia-free survival.

Regulatory T cells (Treg) are a specialized subpopulation of CD4^+^ T cells, characterized by high and constitutive expression of CD25 (interleukin-2 receptor alfa-chain) and the transcription factor Foxp3. Treg cells play an essential role in controlling the immune response and maintaining homeostasis. This is achieved through suppression mechanisms that limit the proliferation and function of other cell types (1–3). Indeed, several studies have shown that both the occurrence and the severity of GVHD are strictly and significantly related to reduced absolute numbers and frequencies of Foxp3^+^CD4^+^CD25^+^ Tregs (4–8). Furthermore, patients receiving grafts with higher numbers of donor Foxp3^+^ Treg display a lower risk of developing GVHD (9–11). Accordingly, therapeutic strategies to reconstitute this population have emerged as a potential alternative treatment for GVHD. Therefore, in order for Treg cells to be used as a safe and effective therapeutical approach to treat GVHD, the suppressive effects of Treg in GVL must be considered, as they would likely lead to disease relapse and treatment failure.

## Considerations on the Treg influence on GVL: lessons from animal models

Initial evidence from animal models suggested that Treg may suppress GVHD while preserving GVL. In 2003, using a mismatched mouse model, Edinger et al. (12) demonstrated that Treg prevent GVHD induction by inhibiting the expansion of alloreactive donor T cells, but not their activation or cytolytic capacity, allowing for the preservation of alloreactive T cells’ ability to eradicate established tumors. In the same year, Jones et al. (13) verified the relevance of controlling the timing of Treg administration, showing that only the very early infusion of expanded Treg regulates severe acute GVHD in an haploidentical mice model. The drawback of this strategy is that it may reduce the chance of sufficient GVL effect to occur. However, a delayed infusion of Treg after GVHD onset would give appropriate time for unrestricted alloreactive responses to be established. To confirm their hypothesis, they tested a less aggressive system, in which mice were matched for the major histocompatibility complex (MHC) but mismatched for minor histocompatibility antigens (mHA). In this model, donor-derived Treg, infused as late as ten days after the transplant/tumor challenge, increased animal survival without compromising the GVL response. In summary, the use of Treg in GHVD prophylaxis or treatment must consider the suppressive mechanisms and the time frame of Treg infusion, so that crucial GVL responses are not hampered.

Although animal studies provide valuable information, the complexity of the GVHD/GVL balance requires studies that explore these mechanisms in humans. In addition, the high diversity of protocols used in mouse models leads to contradictory results. For instance, differences in tumor cells injected in mice can lead to distinctive data that must be examined carefully, as some tumor lines can be easier to eradicate than others. The lack of GVL inhibition reported by Edinger and colleagues when using fresh Treg was tested on A20 and BCL1 cell lines (12). This was confirmed in another study, where the infusion of *ex-vivo* expanded recipient-specific Tregs preserved GVL following the injection of A20 lymphoma cells. However, GVL was not preserved when the P815 mastocytoma cell line was tested using the same GVHD mouse model (14), and also in a different study (15).

To bring mouse models closer to the reality of human tumors, Zhang et al. (16) tested a tumor cell line that is more aggressive and representative of human myeloid leukemia. In this setting, the Treg infusion was found to impair GVL responses by suppressing both responder T cell proliferation and proinflammatory cytokine production. In another study, however, the co-infusion of human Treg and Tcon in mice that had leukemia engraftment by primary human AML cells, SUP-B15 or Burkitt´s cells line showed that Treg did not compromise the anti-tumor effect of Tcon and still avoided GVHD (17). Such discrepancies highlight the importance of considering the nature of the tumors involved, as their biological features, such as aggressiveness and location, will likely influence Treg-mediated GVL suppression, especially in cases where both GVHD and GVL responses are driven by the same mechanisms. This stresses the importance of addressing this issue during pre-clinical Treg tests for GVHD therapy in humans.

## Treg as a prophylactic or therapeutical approach for GVHD and their impact on GVL in human trials

The possibility that Treg impair anti-tumor specific T cell activity is a matter of concern when their clinical usage in GVHD is addressed. While studying immune reconstitution post-HSCT, Nadal et al. (18) have shown that disease-relapsed patients undergoing HSCT for myeloid leukemia had twice the number of peripheral blood (PB) Treg cells in circulation than patients in remission. Indeed, Tregs were the only predictive variable of disease relapse, by logistic regression analysis, in this study. On the other hand, Wolf et al. (19) did not find an association between Treg numbers in the graft and the risk of malignancy relapse in 58 patients in an HLA-matched context. It is therefore important to clarify the impact of Treg-based therapies in the GVHD/GVL balance in the clinical setting.

The promising potential of Treg manipulation to control GVHD has led to clinical trials evaluating Treg infusions before and after GVHD onset, where careful assessment of the impact on GVL was considered. Although Treg infusions result in increased Treg numbers that are essential to prevent or treat GVHD, the preservation of the GVL effect requires the presence of conventional T cells (Tcon) in circulation in the patient. In order to control the Treg : Tcon ratio to sustain the balance between GVHD and GVL responses, some studies tested the co-infusion of donor-Treg and donor-Tcon cells, either in 1:1 (20) or 2:1 Treg to Tcon ratio (17, 21). In one of the first trials, 28 patients undergoing haploidentical HSCT received an early infusion of Tregs four days before the transplant and the Tcon infusion. This regimen prevented GVHD in the absence of any post-transplantation immunosuppression, promoted lymphoid reconstitution and improved immunity to opportunistic pathogens. Furthermore, there was no apparent compromise of the GVL effect, as only one relapse occurred in their high relapse-risk cohort (21). Subsequent studies from the same group expanded the trial to a longer 45-month follow-up, demonstrating once more that infused donor Treg used prophylactically resulted in GVHD suppression without loss of GVL activity, as seen by the very low cumulative incidence of relapse compared to the historical controls (17). The authors’ subsequent trial in 50 patients in a myeloablative conditioning regimen, adapted to the patient’s age, tested the same Treg and Tcon co-infusion immunotherapy showing once again, a low rate (4%) of leukemia relapse (22).

Importantly, the timing of either Treg or Tcon donor cell infusion is relevant (**Table 1**). For instance, in a prophylactic approach, the administration of Treg two to four days before the transplant/Tcon infusion was especially advantageous for GVHD prevention, as it allowed time for a robust expansion of Treg *in vivo* while preserving GVL (17, 21, 26). In that way, this strategy reduces the need to transfer high numbers of Treg cells, which can be difficult to obtain without *in vivo* or *ex vivo* expansion protocols (23, 27). Besides the infusion timing, the clinical trials that explore the use of Treg in GVHD also vary in the source of Treg, in their purification for direct infusion or expansion, and in the level of Foxp3 expression of the product. Such variability in experimental design impacts differently on GVL responses and affects the comparisons across studies, as summarized in **Tables 1** and **2**. It is worth mentioning that fresh polyclonal Treg, *ex vivo* expanded Treg and *in vitro* induced Treg (iTreg) are all different products that should be investigated in specific clinical trials assessing safety and efficacy in each setting.

**Table 1:** The impact of the heterogeneity of adoptive Treg infusions for GVHD prophylaxis on the GVL effect.

Type of transplant	Treg dose and Treg/Tcon ratio	Timing of Treg infusion	Treg source and purification methods	Treg manipulation	Quality control (purity and function)	Outcome of GVHD and GVL effect	Ref.
Haploidentical HSCT	2-4x10^6^/kg Treg; 2:1	Day -4 (CD34^+^ cells and Tcon on day 0)	Magnetic bead selection of CD25^+^ Treg from leukapheresis product depleted of CD8^+^/CD19^+^	Fresh Treg	Mean 69.2% of Foxp3^+^; 67% of *in vitro* suppression in 1:2 Tcon-to-Treg ratio	46.1% of patients were alive and disease-free and one case of relapse after a median follow-up of 12 months. No cGVHD cases	(21)
Haploidentical HSCT	Mean 2.5x10^6^/kg Treg; 2:1	Day -4 (CD34^+^ cells and Tcon on day 0)	Magnetic bead selection of CD25^+^ Treg from leukapheresis product depleted of CD8^+^/CD19^+^	Fresh Treg	Mean 81% of Foxp3^+^; 67% of *in vitro* suppression in 1:2 Tcon-to-Treg ratio	Attenuation of GVHD and significantly lower cumulative incidence of relapse than historical controls	(17)
Haploidentical HSCT	2x10^6^/kg Treg; 2:1	Day -4 (Tcon on day -1 and CD34^+^ cells on day 0)	Magnetic bead selection of CD25^+^ Treg from leukapheresis product depleted of CD8^+^/CD19^+^	Fresh Treg	Mean 71% of CD4^+^CD25^+^CD127^-^Foxp3^+^ cells	48% of GVHD/relapse-free survival after a median follow-up of 29 months	(22)
HLA-matched sibling HSCT	Original protocol: 1x10^6^ Treg/kg; 1:3. Modified protocol: 1-3x10^6^ Treg/kg; 1:1	Day 0 with CD34^+^ (Tcon on day +2)	Magnetic bead selection of CD25^+^ from CD34-depleted apheresis fraction followed by FACS of CD4^+^ CD127^lo^	Original protocol: cryopreserved Treg; Modified protocol: fresh Treg	Median 94% Foxp3^+^; Treg presented *in vitro* suppressive function	Only modified protocol did not cause GVHD. No impairments of GVL despite the high risk of relapse patients	(20)
Partially HLA-matched UCBT	0.1-3x10^6^ UCB Treg/kg (1^st^ infusion) + 3x10^6^ UCB Treg/kg (2^nd^ infusion cohort); no Tcon infusion	Day +1 of UCBT and day +15 for the 2^nd^ infusion cohort	Magnetic bead selection of CD25^+^ from third partially HLA-matched cryopreserved UCB	Expanded *in vitro* with CD3/28 coated beads and IL-2 for 18 days, and cryopreserved for 2^nd^ infusion	Median 64% CD4^+^CD127^-^Foxp3^+^ after expansion; median *in vitro* suppression of 85.5% in 1:4 Tcon-to-Treg ratio	In the highest Treg dose: reduced incidence of II-IV aGVHD (39% vs. 61%); no cGVHD vs. 26%; and cumulative incidence of relapse 23% vs 50% in historical controls	(23)
Partially HLA-matched UCBT	3-100x10^6^ UCB Treg/kg; no Tcon infusion	Day +1 of UCBT	Magnetic bead selection of CD25^+^ from third partially HLA-matched cryopreserved UCB	Expanded *in vitro* with anti-CD3 loaded KT64/86 artificial APCs and IL-2 for 18 days	Median 87% CD4^+^Foxp3^+^ CD127^–^ after expansion; median *in vitro* suppression of 53% in 1:4 Tcon-to-Treg ratio	Reduced II-IV aGVHD incidence (9% vs. 45%) and no cGVHD vs 14% in contemporary control group; similar relapse rates (33% vs. 40% in controls)	(24)
HLA-matched sibling PBSCT	3-300x10^6^/kg iTreg; 1:86, 1:8, 2:1 and 1:1	Day 0, at least 4h before transplant	Magnetic bead depletion of CD25^+^ from apheresis product followed by CD4^+^ beads isolation, for iTreg differentiation	iTreg from CD4^+^CD25^-^ stimulated with anti-CD3 loaded KT64/86 artificial APCs, IL-2, rapamycin, and TGF-ß for 14 days	60% Foxp3^+^ within iTreg product; median *in vitro* suppression of 64% in 1:2 iTreg-to-PBMC ratio	No significant difference in aGVHD, cGVHD and relapse at 2 years cases between iTreg cohort and contemporary controls	(25)

HSCT, hematopoietic stem cell transplantation; UCBT, umbilical cord blood transplantation; HLA, Human leukocyte antigen; Treg, regulatory T cell; Tcon, conventional T cell; PBSCT, Peripheral Blood Stem Cell Transplant; aGVHD, acute graft-versus-host disease; cGVHD, chronic graft-versus-host disease; GVL, graft-versus-leukemia; PBMC, peripheral blood mononuclear cells; KT64/86, K562 cells modified to express CD64 and CD86; iTreg, in vitro induced Treg.

**Table 2:** The impact of the heterogeneity of adoptive Treg infusions for GVHD treatment on the GVL effect.

Type of transplant	Treg dose	Timing of Treg infusion	Treg source and purification methods	Treg manipulation	Quality control (purity and function)	Outcome of GVHD and GVL effect	Ref.
HLA-matched sibling BMT or PBSCT	cGVHD patient: 0.1x10^6^ Treg/kg; aGVHD patient: total of 3x10^6^ Treg/kg	cGVHD patient: 34 months after transplant; aGVHD patient: 3 infusions on day +75, +82, +93 after transplant	CD4^+^ negative immunomagnetic isolation followed by CD4^+^CD25^hi^CD127^-^ FACS from donor leukocyte buffy coat	Expanded *in vitro* with anti-CD3/28 beads and IL-2 for 3 weeks	90% of Foxp3^+^ on 1^st^ infusion, 70% and 40% on 2^nd^ and 3^rd^ infusions when applicable; microbial safety check	Attenuation of disease and tapering of immunosuppression in cGVHD patient; transient improvement of aGVHD with further death from multiorgan dysfunction; no information about relapse	(28)
HLA-matched donor HCT	0.97- 4.45x10^6^ Treg/kg for 1^st^ infusions and one 2^nd^ infusion of 0.52x10^6^ Treg/kg	Median 35 months after transplant	Magnetic bead selection of CD25^+^ from donor leukapheresis product depleted of CD8^+^	Expanded *in vitro* with anti-CD3/28 beads, IL-2, and rapamycin for 12 days; administration of low-dose IL-2 for *in vivo* Treg expansion in 3/5 patients	Median 84.1% of CD4^+^CD25^high^CD127^low^Foxp3^+^; Treg presented *in vitro* suppressive function.	Improvement of clinical response of cGVHD in 2 and stable disease in 3 patients for the initial months, then progressive disease in a patient; reduction of immunosuppression in 3 patients; no relapse of original malignancy, but development of skin cancers in 2 patients	(29)
HLA-matched and HLA-mismatched HSCT	0.1-1x10^6^ Treg/kg	Median 37 months after transplant	Magnetic bead selection of CD25^+^ Treg from leukapheresis product depleted of CD8^+^/CD19^+^	Fresh Treg infused concomitant with administration of low-dose IL-2 for *in vivo* Treg expansion for at least 8 weeks	Median 88.4% CD4^+^CD25^+^CD127^lo^	20% of patients had partial response, 72% had stable disease, 4% had a mixed response, and 4% had progressive disease by week 8. 4-year OS was 65% with no cases of malignant relapses. Immunosuppression was tapered	(27)
HLA-matched donor HSCT	0.5-3x10^6^ Treg/kg	Mean 42 months after cGVHD diagnosis	Magnetic bead selection of CD25^bright^ Treg from leukapheresis product depleted of CD8^+^/CD20^+^	Fresh Treg	Mean 67-73% of CD4^+^CD25^++^CD127^low^Foxp3^+^; Treg presented *in vitro* suppressive function.	70.9% of patients had complete or partial response on cGVHD, and 29% progressed or did not respond, at month 12 after Treg infusion. No cases of relapse.	(30)

HSCT, hematopoietic stem cell transplantation; HCT, hematopoietic cell transplantation; HLA, Human leukocyte antigen; BMT, bone marrow transplantation; Treg, regulatory T cell; PBSCT, Peripheral Blood Stem Cell Transplant; aGVHD, acute graft-versus-host disease; cGVHD, chronic graft-versus-host disease; GVL, graft-versus-leukemia; OS, overall survival.

In the umbilical cord blood (UCB) transplant context, the transfer of the highest dose (3x10^6^) of UCB expanded Treg/kg correlated with reduced aGVHD, no cGVHD cases, and a lower relapse rate than historical controls (23). In a subsequent study, these authors were also able to show that there was no increase in disease relapse as compared to the control group even when high doses of Treg were used (100x10^6^ UCB Treg/kg) (24). However, in this UCB transplant model, Treg were collected from a third-party donor, which likely limited their survival and activity in the patients. Other clinical trials also did not find impairments in the GVL in HLA-matched sibling transplant, either when using an iTreg product, obtained from CD4^+^CD25^-^ precursors, that were further expanded *in vitro* (25), or when using freshly isolated, highly purified sorted Treg followed by a Tcon infusion (20).

While most of the studies focus on the prophylactic administration of Treg to prevent GVHD, some trials have instead investigated the ability of late Treg infusions for GVHD treatment (27–30) (**Table 2**). The rationale behind this approach is based on the knowledge that leukemia is likely to have been eradicated at that point and therefore such studies do not generally prioritize the evaluation of the GVL response. Accordingly, relapse of the original tumor was not observed, although other skin cancers arose in some patients after Treg infusion in one of the studies (29). Thus, in trials using Treg therapeutically in the treatment of severe GVHD, other aspects besides leukemia relapse must be carefully evaluated, such as responses to infections or the emergence of new tumors (29, 31), that may also be hindered by the immunosuppressive activity of Treg.

## Enhancing the GVL effect after Treg infusion for GVHD suppression

In order to decrease the aforementioned risks, the selective expansion of alloantigen-specific Treg (allo-Treg) for GVHD therapy has been pursued, as it generates more suppressive, specialized, and overall efficient Tregs than those that are polyclonally expanded (14, 32–36). Despite the greater specificity of allo-Treg, the possibility remains that some mHA can mediate GVHD but also be involved in GVL responses. For instance, due to H-Y mHA, which is only expressed in males, there is an increased chance of GVHD in male recipients from female HLA-matched donors, but also a reduced risk of relapse in those recipients (37). In mice, H-Y-specific iTreg preserved GVL when infused in mice with pre-established leukemia (36). The same group was able to expand human H-Y-specific Treg *ex vivo*, with the premise that they would prevent GVHD while sparing GVL responses against other mHA such as HA-1 and HA-2 (35, 38). However, the authors have not published to date the results relating to the use of their HY-specific iTreg in a clinical setting (15). Thus, it remains to be clarified whether monoclonal mHA-specific Treg would preserve GVL in humans or not. Likely, the enrichment of donor Treg cells specific to an array of mHA in the healthy tissues of the recipient would provide a more efficient and less broad suppression of alloreactive responses, possibly sparing the GVL effect.

More recently, the use of chimeric antigen receptors (CARs) has been exploited for the generation of antigen-specific Treg to promote transplantation tolerance. This approach likely allows the use of smaller cell doses and reduces the risk of broad and unspecific immune suppression. MacDonald et al. (39) created a CAR Treg specific to one of the most common mismatched antigens, the HLA-A2. Its forced expression on human Tregs promoted stronger proliferation compared to endogenous TCR stimulation, thus allowing the required doses for adoptive therapy to be obtained more easily. Importantly, the HLA-A2-specific CAR-Treg retained their phenotype and stability after *in vitro* expansion and suppressed allo-responses in a xenogeneic mouse model *in vitro* more effectively than polyclonal Treg. Of note, CAR-modified CD4^+^ Tcon can promote antigen-specific lysis as efficiently as CD8^+^ cells and produce high levels of pro-inflammatory cytokines, that can lead to cytokine release syndrome (CRS). Nevertheless, several studies have shown that CAR-Treg does not present a risk for CRS due to the reduced production of inflammatory cytokines after activation (39–41). Regarding cytolysis, although the authors could not find great levels of cytolytic activity in their CAR-modified Tregs in immunodeficient xenogeneic mice (39), such risks still need to be evaluated in humans as HLA-A2 expression is ubiquitous, thus increasing the likelihood of direct tissue damage in HLA-A2^+^ recipients. In fact, another study indicated that CAR-Tregs are able to employ some level of antigen-specific cytotoxicity despite CAR-specificity (42). Therefore, caution should be employed when considering broadly expressed antigens, such as HLA class I molecules, for CAR-Treg constructs, as this may induce a robust CAR stimulation, resulting in a generalized immunosuppressive state that may ultimately impair GVL responses.

With such limitations in mind, other targets started to be investigated for CAR-Treg creation. CD19-directed CAR-T cells have been approved and successfully used for the treatment of B-cell malignancies. Therefore, some studies generated and examined the potential of CD19-targeted CAR-Tregs to suppress B cells while delaying GVHD. Bolivar-Wagers et al. (40) used a fully MHC-mismatched allo-HSCT mouse model to test murine Treg containing a human CD19 CAR construct, that caused B cell aplasia without systemic toxicity. In a mouse model for aGVHD, they observed that in the presence of the human CD19 target, such CAR-Treg suppressed aGVHD efficiently by reducing proinflammatory cytokine-producing by Tcon and increasing Treg in Treg/Tcon ratio especially in the colon, a key target organ in aGVHD. Moreover, such CAR-Treg cells showed antigen-specific killing capacity that depended on perforin but not granzyme B (GZB) production, thus providing direct targeting of CD19^+^ tumor cells and reducing lymphoma cell growth *in vivo* (40). On the other hand, another study engineered CD19 CAR-Treg from human CD45RA^+^ purified Treg and used the CD28 costimulatory domain instead of 4-1BB (41), as in the previous study. In this case, CD45RA^+^ CD19 CAR-Treg did not present cytolytic activity, although they were able to suppress human B cell Ig production and differentiation into plasma cells in a xenogeneic mouse model of GVHD, also leading to attenuation of the disease.

These studies indicate that CAR-Treg administration has the potential to suppress GVHD while maintaining sufficient antitumor response. More recently, a clinical trial using a CD6-CAR Treg in GVHD was launched, aiming to take advantage of CD6-targeted anti-inflammatory response (clinicaltrials.gov identifier: NCT05993611). Noteworthy, it is important to acknowledge the differences in targets and costimulatory domains used in CAR construct, as they will dramatically impact the final product and therefore the effect and safety of the therapy (41, 42). Overall, more preclinical studies and the development of novel molecular on/off switches on CAR-Treg are important for the advancement of this strategy for GVHD therapy while preserving GVL (43).

Besides genetic modifications of the classical Foxp3^+^ Treg population, some authors are exploring the clinical potential of distinct regulatory subsets, such as type 1 regulatory (Tr1) cells. Tr1 are peripherally generated cells with suppressor ability associated with high IL-10 production, in the absence of Foxp3 expression. It has been shown that IL-10-engineered human CD4^+^ resemble natural Tr1s and present direct GZB-mediated cytotoxicity against myeloid leukemic cell lines, in an HLA class I-dependent manner, regardless of TCR specificity (44). In different humanized mouse models, these cells suppressed xenogeneic GVHD while preventing leukemia development by mediating directly anti-tumor effects (44). In a subsequent study, the same authors observed that engineered Tr1 efficiently killed pediatric AML cell samples *in vitro*, suggesting that the adoptive transfer of such Tr1 could be performed alongside allo-HSCT, to prevent GVHD (45). In a Phase I clinical trial, the authors infused their IL-10-engineered donor T cell product in 12 patients. Despite achieving only partial control of GVHD, likely because of the small percentage of Tr1 cells in the product, 4 patients who attained immune reconstitution remained relapse-free for a median follow-up of 7.2 years (46). Currently, optimizations in the protocols for *in vitro* generation of Tr1 cells are being performed in order to increase Tr1 number and efficacy of the infusion product (47).

Other strategies are further being adopted that aim to enhance GVL after classical Foxp3^+^ Treg infusion. For instance, the co-administration of Treg with different substances, such as ruxolitinib, low-dose IL-2, IL-33, and rapamycin, are being tested. In fact, the effect of low-dose IL-2 after HSCT has been studied for decades, demonstrating the ability of this cytokine to modulate GVHD without impairing GVL (48–51). In recent clinical trials, the use of low-dose IL-2 prophylactically (52) or therapeutically in steroid-refractory cGVHD patients (53) did not impair GVL. Of note, in the latter case, low-dose IL-2 induced a preferential increase in Treg cell counts (53). Given such results, another trial combined low-dose IL-2 with donor-derived Treg cell therapy seeking to induce greater Treg expansion *in vivo*. Again, GVL was not abrogated since no relapse cases were reported. However, clinical improvement was only observed in 20% of patients, likely due to the advanced phase of cGVHD and to the low number of donor-Treg infused (27).

Using a mouse model, Meguri et al. (54) have shown that IL-2 therapy affects Treg and effector T cells (Teff) responses differently depending on the immune environment in the host. In mild inflammatory conditions, the IL-2 therapy controlled GVHD without affecting GVL. Moreover, their results indicate that in an immune-tolerant state after HSCT, IL-2 therapy may even enhance the GVL effect without exacerbating GVHD (54). More recently, adaptations to the previous approaches using IL-2 therapy have been performed aiming to specifically promote Treg expansion and not alloreactive effector T cells. For instance, orthogonal IL-2 specifically binds the ortho IL-2 receptor β-chain, which in turn was forced expressed on mouse Treg. These engineered Treg cells selectively expanded *in vitro* and *in vivo* in the presence of ortho IL-2 in an MHC-mismatched mouse model and were capable of suppressing aGVHD while maintaining GVL responses against A20 and MLL-AF9 cells, even when low cell numbers were infused (55). The authors suggest combining early Treg infusion (i.e. before Tcon in HSCT (20, 26)) with their orthogonal system to support Treg expansion *in vivo* (55). Another alternative improvement to the IL-2-based therapy is through the short administration of IL-2/anti-IL-2 complexes, which can be modulated to preferentially induce Treg over Teff cells. Thiolat et al. (56) found different benefits in this approach, such as prevention of GVHD development and reduction in leukemia-related death in mice, which is likely associated with the significant reduction of exhausted CD8^+^ T cell levels, in a mechanism that is partially mediated by CTLA-4.

Rapamycin (RAPA) selectively inhibits the mammalian target of rapamycin (mTOR), which affects T cell activation, proliferation, and differentiation, while preserving Treg function in which the mTOR pathway is constitutively inhibited (57). In NSG mice transplanted with human peripheral blood mononuclear cells (PBMC) and receiving RAPA daily for three weeks, GVHD was alleviated, and survival rates increased in comparison to the control group, that did not receive the inhibitor (58). RAPA decreased CD4^+^ and CD8^+^ T cell proliferation and apoptosis. Importantly, RAPA administration preserved GVL against the AML THP-1 line in primary and secondary transplants, where an increase in Treg proliferation was observed. In GVHD patients, RAPA treatment inhibits T cell proliferation, mainly in CD8^+^ T cells. RAPA further augments the anti-apoptotic protein BCL-2, Treg counts and CD25 expression in all T cell subsets. Such general increase of CD25 expression induced by RAPA poses a limitation in this approach when low-dose IL-2 therapy is considered, as it relies on the selectively high expression levels of CD25 Treg to expand Treg *in vivo* (58). Noteworthy, RAPA may also directly inhibit leukemia growth through mTOR inhibition. This is shown in a mouse model, where Zhang and colleagues observed that the concurrent administration of RAPA and IL-2 delayed the onset of leukemia (16).

Ruxolitinib is a Janus kinase (JAK) inhibitor with anti-inflammatory properties currently used in steroid-refractory acute and chronic GVHD. Studies in mice and humans suggested that ruxolitinib treatment *per se* is not associated with a higher risk of relapse compared to other immunosuppressors (59–62). Moreover, the combined treatment of ruxolitinib and human Treg administration in a mouse model suppressed GVHD without hampering GVL, despite the preferential activity of ruxolitinib in Treg over Tcon *in vitro* (63). Based on these results, the same group launched a clinical trial using donor Treg infusion in the treatment of cGVHD in patients with no improvement or partial responses to ruxolitinib (30).

Posttransplantation cyclophosphamide (PTCy) is frequently used as GVHD prophylaxis to induce tolerance after HLA-mismatched and HLA-matched allo-HSCT, minimizing the need for additional immunosuppression (64). PTCy mechanism of action was first associated with clonal deletion of early stimulated alloreactive T cells soon after the engraftment in mouse models. More recently, it was proposed that alloreactive T cells are not eliminated, but their expansion is constrained immediately after PTCy administration, while GVL responses occur afterwards (65). Moreover, the PTCy-mediated tolerance induction process seems to be dependent on donor Treg cells, as mice depleted of Foxp3^+^ Treg either before or after PTCy treatment exhibited accelerated acute GVHD development (66). However, studies in humanized mouse models did not find the presence of Treg mandatory for PTCy-induced GVHD inhibition, as PTCy significantly mitigated the xenogeneic GVHD even when human PBMC depleted of CD25^+^ cells were infused (67). On the other hand, mice receiving untouched PBMC showed improved survival rates than those in which Treg were depleted. Moreover, when untouched were used, GVL was decreased, but not abrogated (67). Overall, those studies point to an indispensable and nonredundant role for Treg in PTCy activity, which in turn does not disturb the GVL effect.

Noteworthy, studies in human allo-HSCT demonstrated that Treg cells recovered faster than Tcon after PTCy therapy, due to a higher expression of aldehyde dehydrogenase (ALDH) in Treg than in Tcon that seems to provide the former increased resistance to Cy, since ALDH is a major mechanism of Cy inactivation *in vivo* (68). In fact, analysis of patients undergoing allo-HSCT and PTCy have shown higher frequency of Treg cells 30 days after transplant compared to patients without PTCy. CD8^+^ T cells in five out of 23 patients who relapsed expressed less GZB and perforin than CD8^+^ T cells from patients in remission for a median follow-up of 13.1 months, suggesting a positive correlation between these markers and GVL (69). Similar to PTCy, azacytidine has been used to reduce GVHD without impairing GVL. Azacytidine also suppresses effector T cell proliferation but not Treg. Post-transplant azacytidine induced an increase in Treg cell numbers while promoting a CD8^+^ T-cell cytotoxic response in AML patients receiving three treatment cycles (70). In mice, azacytidine was able to convert Teff into T cells with a regulatory phenotype through hypomethylation of the Foxp3 promoter, resulting in augmented Foxp3 expression (71). As the correlation between Treg, GVL, PTCy or azacytidine has been explored more recently, additional studies are needed to unravel such complex associations.

To clarify the issue of Treg to Teff ratios in the GVHD/GVL balance, a recent study used a computational model to explore the bidirectional molecular interactions between Tregs and Teffs in allo-HSCT, as a core regulatory network that may be used as a strategy to enhance the GVL effect (72). The model predicted shifts in Tregs and Teffs numbers upon simultaneous blockade of CD25, TNFR2 and CTLA-4, suggesting this would favor GVL after allo-HSCT without causing GVHD. However, this model requires clinical testing (72). In fact, mouse studies in HLA-mismatched and matched settings have shown that the modulation of another member of the TNF receptor superfamily the TNFRSF25, alongside CD25 pathway, promotes the expansion of Treg *in vivo*. Treg up-regulated activation markers and showed enhanced suppressive function, that ameliorated GVHD while preserving GVL (73, 74). A subsequent study from the same group tested a mouse model of transplant that combines mobilized PB and *in vivo* Treg expansion by the TNFRSF25/CD25 pathway, in which the expanded Treg were essential for mediating GVHD suppression and did not interfere with GVL (75).

Alongside the use of therapeutic drugs that promote Treg expansion *in vivo* while preserving GVL, alternative cell-based approaches that use other suppressor cell populations are under investigation. In this setting, myeloid-derived suppressor cells (MDSC) have been shown to induce Treg cells *in vivo* and increase their suppressive function, while avoiding GVHD and maintaining GVL in mice (76, 77). An increasing body of evidence further shows that CD8^+^ Treg cells are able to attenuate GVHD and possess tumor-killing features (78, 79), raising the possibility of an approach consisting of the combined infusion of CD4^+^ and CD8^+^ Tregs in the future, as suggested by some authors (15, 80).

IL-33 is an IL-1 family member that is released upon tissue damage, inducing the activation of MDSC and Treg cells (81, 82). Indeed, in a mouse model of HSCT, Treg expanded *ex vivo* with IL-33 protected mice from GVHD in an amphiregulin (AREG) mediated way (83). In another study, *in vivo* administration of IL-33 from day -10 to 4 days after HSCT induced the expansion of ST2^+^ Treg, that were resistant to total body irradiation. In turn, those cells reduced effector T cell levels and controlled IL-33-driven aGVHD (84). Importantly, the blockade of the decoy IL-33 receptor, the soluble form of ST2, allowed free IL-33 to interact with membrane-bound ST2 expressed on Treg. This led to an increase in Treg frequency to the detriment of pathogenic Th17 cells, thus controlling GVHD in a mouse model. Nevertheless, the *in vitro* anti-tumoral cytotoxicity and the *in vivo* GVL activity were preserved, presumably because other CD4^+^ T cell subsets were less affected by the blockade in ST2 (85). IL-33 also promotes the expansion of a subset of IL-9-producing T cells that contribute to GVHD prevention. This is achieved through AREG production, thus maintaining effector T cell function, and preserving GVL (86).

## Possible mechanisms by which GVL may be maintained upon adoptive Treg transfer for GVHD therapy

**Figure 1:**
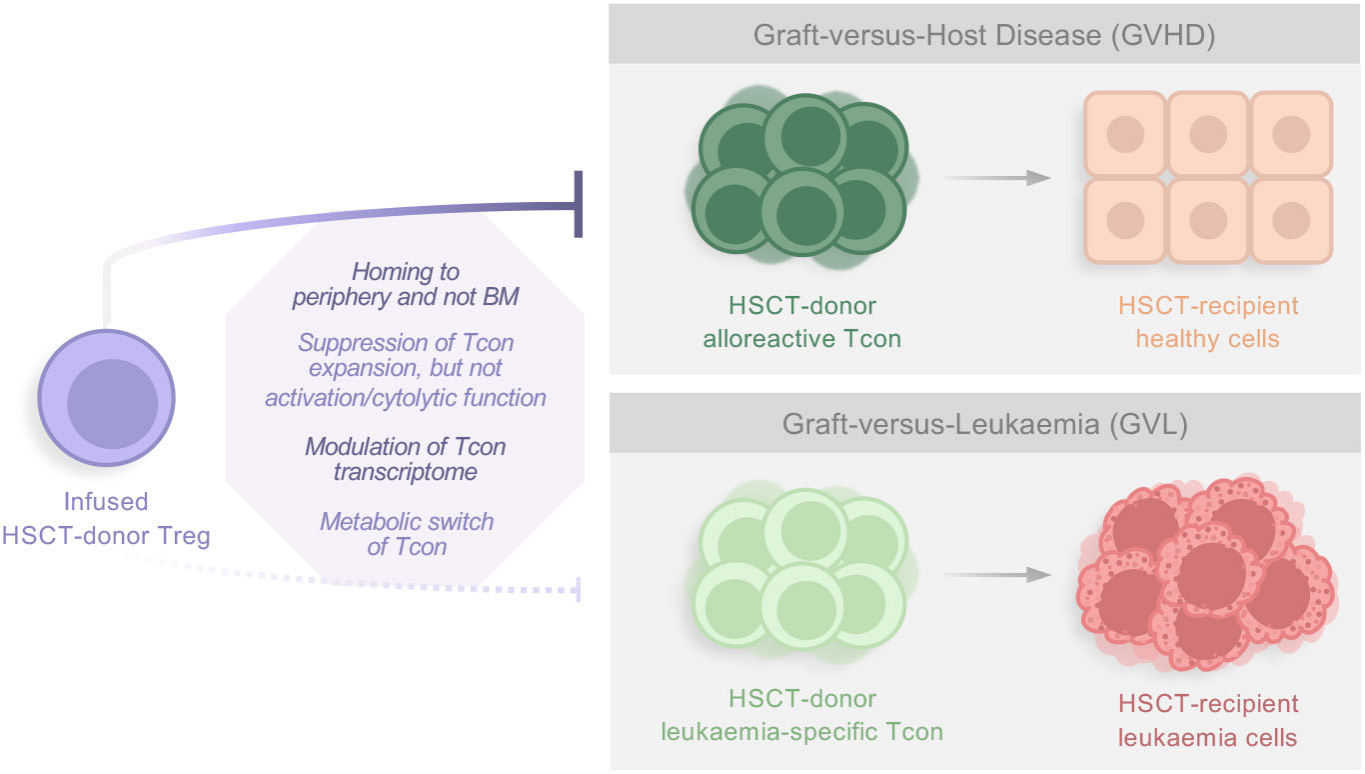
Factors influencing Treg suppressive activity against GVHD while preserving the GVL effect. Studies have shown that several parameters seem to be responsible for the ability of infused donor-Treg to suppress alloreactive Tcon responses causing GVHD, while not interfering with leukemia-specific Tcon, and thus maintaining GVL. In general, reasons include homing discrepancies, which suppression mechanisms are involved, and their targets. HSCT, Hematopoietic Stem Cell Transplantation; Treg, regulatory T cell; BM, blood marrow; Tcon, conventional T cell.

The reasons behind the ability of Treg to suppress allo-specific responses causing GVHD, while maintaining GVL, have been increasingly investigated and pointed out to homing discrepancies, the suppression mechanisms involved and their targets (**Figure 1**). Regarding the former, human PB Tregs preferentially activate at lymph nodes and migrate to GVHD target organs, such as skin, gut, liver, and lungs, but not to the blood marrow (BM) (26, 87). Thus, one possible theory postulates that GVHD suppression by Treg and the alloreactive T cell responses that control leukemia occurs in distinct tissues. In fact, most of the Treg present in human PB express CD45RO and low levels of CXCR4. This implies that once infused, such Treg are likely unable to home to the BM, restricting their suppressive activity to the periphery. This likely results in decreased GVHD while allowing Teff alloreactivity in the BM. This was tested in NSG mice receiving human leukemia, Treg and Tcon cells. These mice survived without cancer or GVHD, while this did not happen when the injected Treg expressed CD45RA and CXCR4. In that case, functional infused Tregs were found in the BM and mice died from the tumor progression, even though large doses of Tcon were given. When CXCR4 was blocked in this setting, however, Treg migration to the BM did not occur and GVL was preserved (88). Thus, these results suggest that infused Treg in the aforementioned clinical trials might not interfere with GVL due to their inability to migrate to the BM while suppressing GVHD responses that occur in the periphery.

Recently, the same group analyzed human PB and BM samples at different time points after Treg/Tcon immunotherapy in haploidentical transplantation. Tolerogenic dendritic cells were found in PB, while pro-inflammatory dendritic cells and CD161^+^ Treg were detected in the BM. *In vitro* studies using induced CD161^+^ Treg isolated from healthy donors indicated that these cells favor Tcon-mediated killing capacity (89). As CD161^+^ Treg cells have been described to produce pro-inflammatory cytokines while still maintaining suppressor phenotype, it is possible that this cell population can even play a permissive role in GVL *in vivo* while actively participating in the anti-tumor effect. Such observations demand further investigation. Finally, the authors suggest that GVL is maintained due to the preferential migration of infused Treg to the periphery during the initial post-transplantation period, while the development of CD161^+^ Treg in the BM may contribute to the GVL effect afterwards (89).

Other studies have further shown that Tregs markedly suppress the expansion of alloreactive Tcon clones, but do not interfere with their activation, cytolytic function (12), differentiation or TCR repertoire (90). It was recently shown in a mouse model of MHC-mismatched allo-HCT, that infused Tregs are capable of modulating both pro- and anti-inflammatory gene transcription in both CD4^+^ and CD8^+^ Tcon, leaving the induction of GVL-related genes unaffected. Furthermore, this transcriptomics study indicated that Treg promotes a switch of Tcon metabolic activity from glycolysis to oxidative phosphorylation (90). Importantly, metabolic reprogramming has been studied as a strategy to maintain GVL (91, 92). Overall, these studies suggest possible mechanisms that may explain the lack of interference of infused Treg in GVL in the aforementioned human clinical trials. However, future trials should conduct more analyses in relapsed patients to rule out an effect of the infused Treg in this outcome.

## Conclusions and future perspectives

In the past decade, several studies in humans and animal models have attempted to determine the impact of Treg infusions to treat GVHD on tumor relapse. The evidence gathered appears to suggest that the infusion of donor Treg does not interfere with the ability of donor T cells to clear tumor cells, thus preserving GVL. More studies are required to determine the therapeutic efficacy of such infusions. Noteworthy, the development of more potent Treg must consider the ability of these cells to spare leukemia-specific immune responses. Therefore, it is fundamental that future work in this field aims to develop improved sources of Treg for infusion while concomitantly assessing their suppressive activity against GVHD responses while preserving GVL.

## Author contributions

CP: Writing – original draft. MS: Writing – review & editing. JL: Writing – review & editing.
